# Influence of Aspect Ratio on the Flexural and Buckling Behavior of an Aluminium Sandwich Composite: A Numerical and Experimental Approach

**DOI:** 10.3390/ma16196544

**Published:** 2023-10-03

**Authors:** Ganesh Radhakrishnan, Daniel Breaz, Al Haitham Mohammed Sulaiman Al Hattali, Al Muntaser Nasser Al Yahyai, Al Muntaser Nasser Omar Al Riyami, Al Muatasim Dawood Al Hadhrami, Kadhavoor R. Karthikeyan

**Affiliations:** 1 Department of Mechanical Engineering, College of Engineering & Technology, University of Technology & Applied Sciences, Nizwa P.O. Box 477, Oman; ganesh.radhakrishnan@nct.edu.om (G.R.); s22s161230@nct.edu.om (A.H.M.S.A.H.); s72s179@nct.edu.om (A.M.N.A.Y.); 21s13124@nct.edu.om (A.M.N.O.A.R.); s22s1775@nct.edu.om (A.M.D.A.H.); 2 Department of Mathematics, “1 Decembrie 1918” University of Alba Iulia, 510009 Alba Iulia, Romania; 3 Department of Applied Mathematics and Science, National University of Science & Technology, Muscat P.O. Box 620, Oman

**Keywords:** sandwich composite, laminate, aspect ratio, flexural strength, buckling, inter-laminar strength

## Abstract

In the field of engineering materials, lightweight and ultra-lightweight composites are used in real time to a greater extent, with high-performance targeting for tailor-made systems in aerospace, automotive, and biomedical applications. Sandwich composites are among the most popular lightweight materials used in structural and vehicle-building applications. In the present investigation, one such sandwich composite laminate composed of aluminum face sheets and a high-density polyethylene core was considered to analyze sandwich composites’ flexural and buckling behavior experimentally and numerically. The influence of aspect ratios, such as length to thickness and width to thickness, on the flexural and buckling performance of sandwich composite laminates was explored in the study. Laminates with different widths, namely, 10, 12, and 15 mm, and a uniform thickness and length of 3 mm and 150 mm, respectively, were used for flexural analysis, whereas laminates with widths of 10, 12, and 15 mm and a uniform thickness and length of 3 mm and 350 mm, respectively, were used for buckling analysis. The geometrical influence of the laminates on mechanical performance was studied through performance measures such as critical bending load, flexural stiffness, inter-laminar shear stress, and critical buckling load. A significant influence of aspect ratio on the mechanical behavior of the laminates was observed using both experimental and numerical approaches. Flexural behavior was observed to be better at greater widths, namely, 15 mm, and with a minimum support span of 90 mm due to reduced spring back effects and increased bending resistance. A maximum width of 15 mm allowed for a higher buckling load capacity similar to that of bending resistance. A critical buckling load of 655.8 N seemed to be the maximum and was obtained for the highest aspect ratio, b/t = 5. The soft core and ductile metal face sheets offered combined resistance to both bending and buckling. A lower aspect ratio (span to thickness) rendered these sandwich laminates better in terms of both bending and buckling.

## 1. Introduction

In general, composite materials have revolutionized the materials field in terms of unique, tailor-made features and high performance. Every day, new classes of composite materials are being developed all over the world, with attractive features such as being ultra-lightweight, having improved mechanical and thermal performance, etc. One such popular category of lightweight composite materials is sandwich composites. Sandwich composite laminates are a kind of composite material wherein metal–polymer–metal sandwich sheets are bonded together with an adhesive. These composites consist of two thin, lightweight metal face sheets and a thick polymer core. The polymer core has low strength, but its high thickness results in higher flexural resistance with low density. The most popularly used core materials include polyurethane, polyethylene, polystyrene, honeycombs, etc. Thermoplastics, thermoset polymers, and sheet metals are used as skin materials. The important feature of a sandwich composite is that its exterior surfaces resist the loads caused by bending or compression, whereas the inner core material resists the load caused by shearing. Basically, sandwich composite structures are types of anisotropic materials; therefore, the strength of the material depends on the nature of the applied load. The selection of materials and their dimensions govern an object’s resistance capability against loading. Sandwich composite structures are mainly used for applications where stiffness is considered a significant factor. Analyzing the mechanical behavior of sandwich composite structures with respect to the components used in the structure and the relevant loading conditions will reveal their significant influence on performance when applied to a real time environment. The properties of the materials used for the core and face sheets and their dimensions have a great influence on the performance of a composite. Simulated analysis of the mechanical behavior of these sandwich composites gives the opportunity to find an appropriate material and determine its usefulness.

In the present work, aluminum sandwich composite (ASC) laminates were selected due to their availability and high performance in real-time applications. Aluminum sandwich composite laminates are used in structural and building applications regarding doors, covering external structures, etc., to provide weather resistance and soundproofing [1,2,3]. They have been investigated for their performance against static flexural and buckling loading. Flexural and buckling behaviors were chosen because failures in structural and building applications are more critical due to bending and buckling. Shear between the face sheets and the core material was observed due to a higher order of bending under transverse loading and lateral buckling under a compressive load [4,5]. The simulated flexural and buckling behavior of the ASC laminate was compared with experimental work to confirm the consistency and accuracy of the results obtained. The key factor, the aspect ratio, which is the ratio between two dimensions of a work piece material, is considered in the flexural behavior study of ASC laminates. Aspect ratios have a significant influence on the flexural behavior of composite laminates. In this study, aspect ratios, i.e., the ratios of width to thickness and length to thickness, are considered and investigated to determine their influence. Ali Isiktas et al. [6] analyzed the cracks on carbon-fiber-reinforced Al laminate formed during bending and found that the dimensions of the cracks increased with the increase in the thickness of the laminate. Similar observations were made by Tang et al. [7] while assessing the bending performance of carbon fiber epoxy laminate. Cracks with slips appeared on the surface of sandwich laminate due to heterogeneity in construction. PVC foam glass-fiber-reinforced polymer laminate was subjected to transverse loading, and multiple failure modes were explored, such as fiber failure, shear failure between the matrix and the fiber, delamination, debonding at the interface, and foam failure. A few years back, Davies [8] reviewed the structural design of elements in the sandwich panel, which consist of two metal faces separated by a lightweight core. The buckling behavior of the panel was investigated in the study through classical and numerical approaches, and the results showed that a greater contribution of resistance to buckling was made by the core material compared to the metal faces. The simplification of the design aspects changed the investigation from a quite complex analysis to an analysis with appropriate results. A numerical analysis of sandwich panels with a new type of element was proposed by Ya Ou et al. [9]. The element they used comprised two face layers connected by another layer. The face layer was considered to serve as a beam, and the intermediate layer was considered to serve as a spring. Researchers and academicians working in the field of sandwich composite structures have contributed many useful outcomes, but there is still a wide scope to pursue with respect to research in this area in order to find novel material combinations, thereby making the resulting product suitable for a desired application. Most of the contributions witnessed in the area of investigation have concentrated on natural- or synthetic-fiber-reinforced tailor-made polymeric composites, and very few research outcomes regarding commercially available sandwich panels have been published. The highlights of this work include the investigation of the bending and buckling behavior of commercially available aluminum sandwich composite laminates in order to recommend them for structural applications, where high specific performance is a desired factor. The novelty of this work is that it considers a geometrical factor, the aspect ratio, as a variable for comparison with other different characterization parameters analyzed in the study. Based on a detailed literature review, it was assumed in the investigation that there was perfect bonding between the face sheets and the core. It was also assumed that there was no delamination between the layers. The metal face sheets were assumed to be elastic at all times. The research objective of this study was to investigate the influence of the aspect ratio of sandwich laminates on their flexural and buckling performance, which, in turn, would help us to arrive at optimized dimensions of the laminates. This objective was chosen to determine how to limit the wastage of a material and thereby increase its specific strength.

## 2. Experimentation

### 2.1. Materials

**Figure 1:**
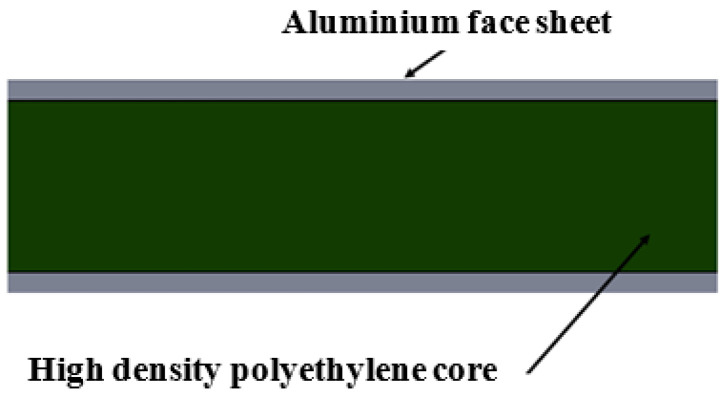
Schematic sketch of the cross section of sandwich composite laminate.

Commercially available aluminum sandwich composite (ASC) laminates with a 2.4 mm thick, black, high-density polyethylene core and aluminum sheet metal pieces of 0.3 mm thick placed on either side of the core as a face material were used in this study. The ASC laminate specimens prepared for three-point bending test had rectangular cross-sections with three different widths corresponding to 10, 12, and 15 mm and a fixed length and thickness of 150 mm and 3 mm, respectively. A schematic sketch of the specimen’s cross section is shown in Figure 1. The dimensions of the specimen were chosen based on the sizes of different types of commercially available aluminum sandwich panels available on the market. The dimensions suitably matched the experimental set up.

### 2.2. Methodology

#### 2.2.1. Experimental Analysis

**Figure 2:**
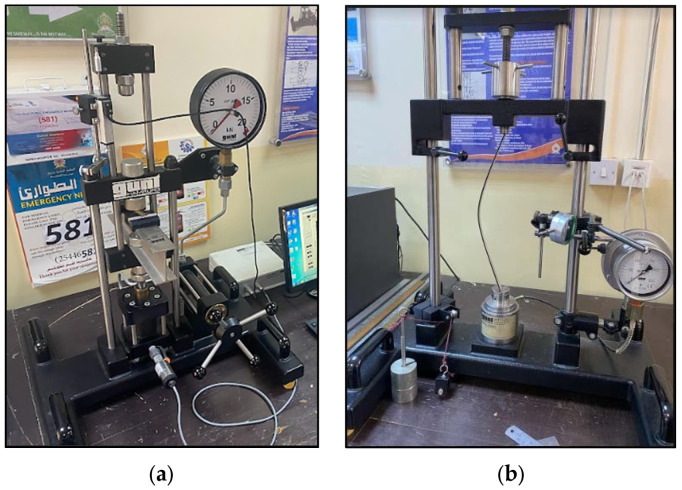
Experimental set up for (**a**) flexural test and (**b**) buckling test.

The flexural behavior of the laminate was tested using a universal Testing Machine (UTM) with a 20 kN capacity supplied by Gunt Hamburg, Brighton, Germany. A concentrated load was applied on the specimen manually at the mid span in slow and gradual increments. The data acquisition system of the machine displayed the deflection versus load as output, from which maximum bending load was estimated. Each experiment was repeated thrice to confirm the accuracy and consistency of the results. This procedure reduces the number of errors that can occur during experimentation. The experimental setup used in the study is shown in Figure 2a,b. The influence of aspect ratio on the flexural behavior of the sandwich laminate was investigated in the study. Aluminum sandwich composite (ASC) laminates were also subjected to buckling test under various end conditions in a buckling behavior machine with a capacity of 2500 N (supplier: Gunt Hamburg, Brighton, Germany). The specimens were prepared with three different widths, namely, 15, 12, and 10 mm, and had a common length of 350 mm. Buckling tests were carried out on the specimens to determine the end conditions based on Euler theory. The specimens were prepared accordingly for the end conditions: pinned–pinned, pinned–fixed, and fixed–fixed. Each test was repeated three times with different specimens to ensure the consistency of results obtained. The output measures such as maximum buckling load and corresponding deflection were noted.

#### 2.2.2. Numerical Analysis

**Figure 3:**
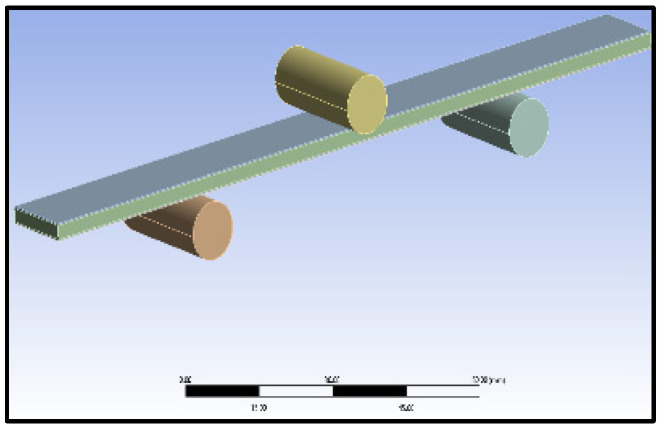
Flexural test model for numerical analysis.

The numerical study model is shown in Figure 3. Since the specimens were very thin and lightweight, the buckling load was observed to be very minimum. In the numerical analysis, the flexural and buckling experiments were modeled using SOLIDWORKS (Version 2018, Dassault Systems, Waltham, MA, USA), with details closer to the experimentation, and imported to ANSYS (ANSYS workbench 2012, ANSYS Inc., Canonsburg, PA, USA). The aluminum sandwich composite laminate was modelled in ANSYS as a single component with three layers across the width, namely, a polyethylene core in the middle that was 2.4 mm thick and aluminum face sheets on either side that were 0.3 mm thick each. All the constituents were assumed to be cohesive in the numerical analysis. In the numerical analysis, the contact between the contacting surfaces, such as the beam, being a sandwich laminate, and the supporting and loading rollers, being made of steel, was considered frictionless. The boundary conditions and constraints were applied in the numerical analysis, matching the conditions applied during the experimentation. The cross-section was symmetrical, and the loading plane was also a symmetrical section. The loading plane varied with respect to the width of the laminate, that is, 10, 12, and 15 mm, whereas the length was maintained constant at 150 mm. The supports were simply supported, with ‘Z’ translation free and ‘X’ rotation free. Fine mesh with standard 10-node tetrahedron elements with a size of 1 mm was used in the numerical analysis for both core and face sheets in order to reduce the computational effort. Constant mesh density was used in the analysis for better resolution. The material properties of the core and face layers were used in the numerical analysis, and they were the same as those used in experimentation. The adhesive interfaces between the metal face layer and polymer core were modelled in ANSYS as cohesive contact elements with zero thickness. The team of Massimo et al. [10] made a similar assumption and followed a similar procedure in their study. Also, nonlinear analysis of the structures with implemented geometric imperfections was conducted by Pawel [11] to study the effect of compression load eccentricities on buckling behavior, whereas in this study, an axial compressive load was applied instead of eccentric load.

## 3. Results and Discussions

**Figure 4:**
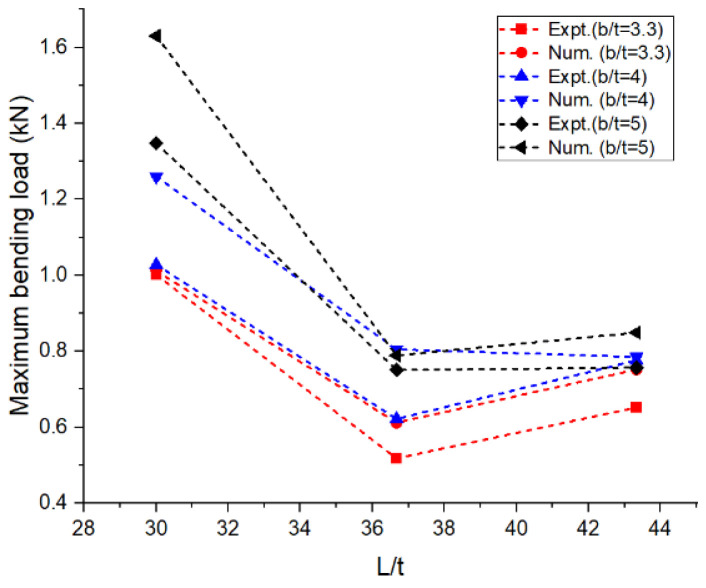
Aspect ratio vs. bending load: numerical and experimental.

**Figure 5:**
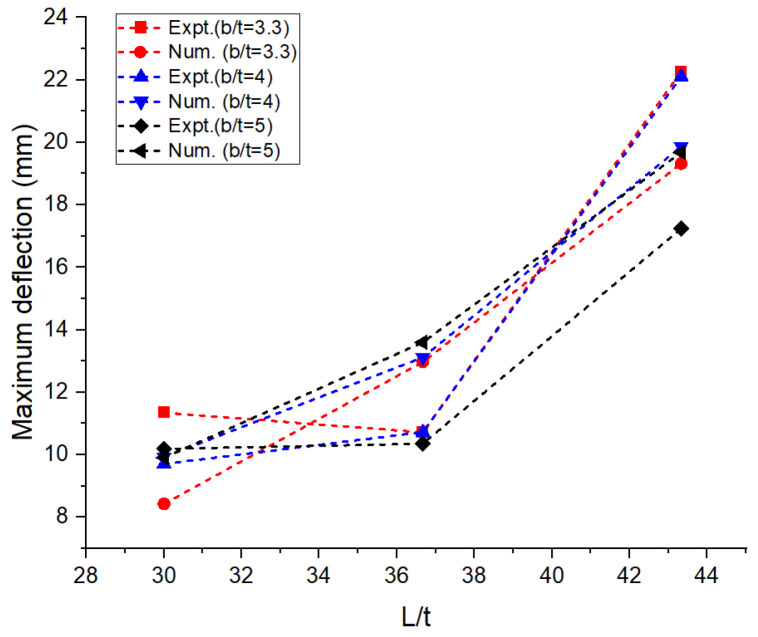
Aspect ratio vs. deflection: numerical and experimental.

**Figure 6:**
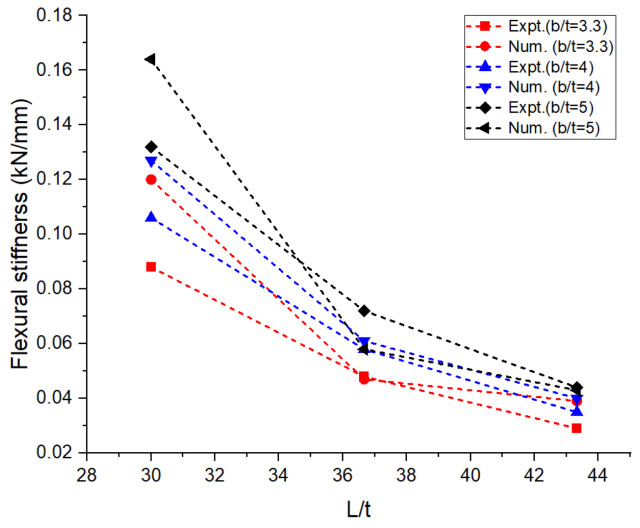
Aspect ratio vs. flexural stiffness: numerical and experimental.

**Figure 7:**
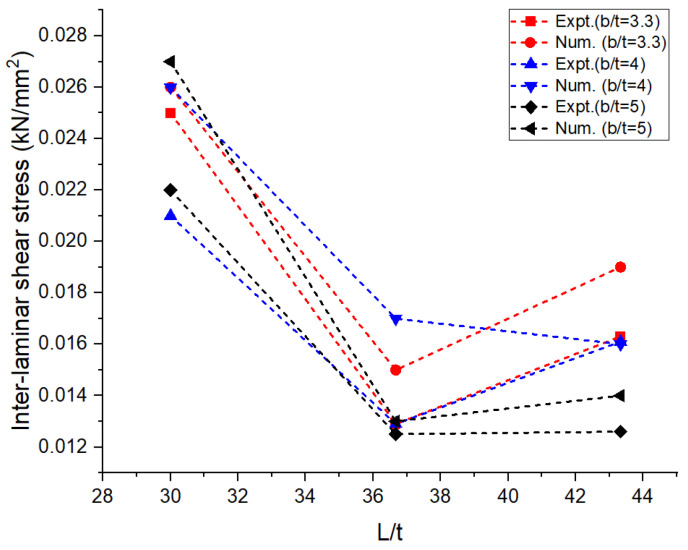
Aspect ratio vs. inter-laminar shear stress: numerical and experimental.

The experimental and numerical values of the performance measures obtained are shown in Table 1. The average values of the performance measures obtained in all the trials are presented in the table. The bending load was maximum at around 1.5 kN for the aspect ratios L/t and b/t corresponding to 30 and 5, respectively. It can be observed in Figure 4 that there was a drastic drop in bending load with an increase in the aspect ratio, L/t, from 30 to 36.67 irrespective of the width of the laminates. On the other side, when L/t increased from 36.67 to 43.33, there was a marginal or negligible rise in the bending load. The greater the width of the laminate, the higher the resistance offered by the specimen against bending [12]. This was achieved through the combined resistance of the polyethylene core and the aluminum face sheets. The thickness of the core used in the construction of the laminate was 2.4 mm, and the thickness of the face sheets used on either side was 0.3 mm. The greater the thickness of the core, the greater the resistance offered against deformation. Figure 5 reveals that the deflection measured at the midpoint of the laminate during the flexural test increased steadily with the increase in the aspect ratio, L/t, from 30 to 43.33. An increase in the support span led to a decrease in the stability of the laminates against transverse bending load irrespective of the width of the laminates [13,14,15,16]. The lowest order of deflection was observed for the wider laminate due to the higher modulus offering higher resistance to bending. The flexural stiffness of the sandwich composite laminates was measured to study the resistance offered by the laminate during deformation and plotted against the support span; this information is shown in Figure 6. The observations regarding the flexural stiffness of the laminates confirmed that the flexural behavior was similar to that obtained through the measurement of deflection. A steady drop in flexural stiffness with an increase in support span was noticed. Since this study focused on a sandwich composite laminate, the important characteristic that needed to be considered was inter-laminar shear stress due to the adhesive contact surfaces available on both sides of the laminate [17,18,19,20]. These adhesive layers often tend to tear off, increasing the possibility of failure. The variation in inter-laminar shear stress against support span is shown in Figure 7. It was observed that there was a steady drop in inter-laminar shear stress as the support span increased from 90 to 110 mm and further increased to 130 mm; the variation in inter-laminar shear stress was very marginal and constituted a negligible quantity. The shear deformation was enhanced significantly with an increase in the support span. This may be attributed to the increased slope of the laminates under loading and the increase in support span. The constituents of the sandwich composite laminate, such as the soft polyethylene core and the metal face sheets on either side, restricted the shear deformation when the support span increased beyond 110 mm. The adhesive layer connecting the face sheet and the core on either side influenced the behavior over the entire span of the laminate [21,22,23].

**Figure 8:**
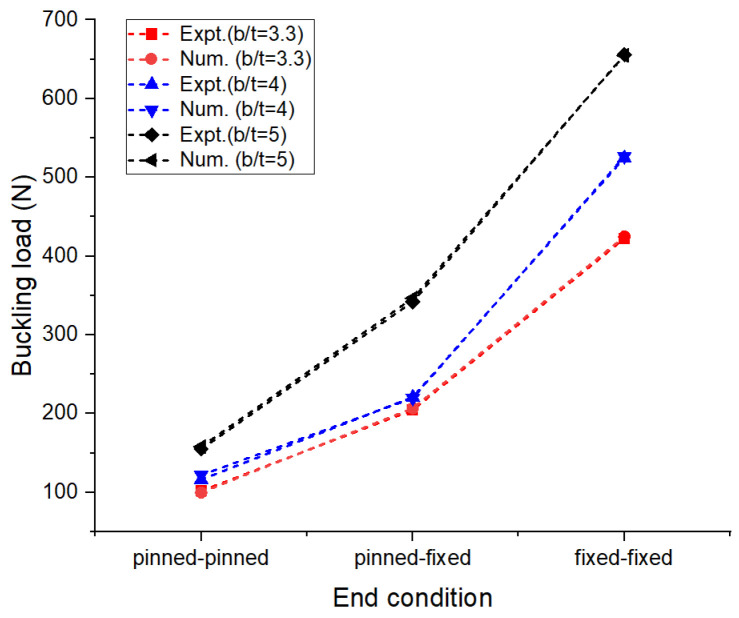
Effect of aspect ratio on buckling load: numerical and experimental results.

**Figure 9:**
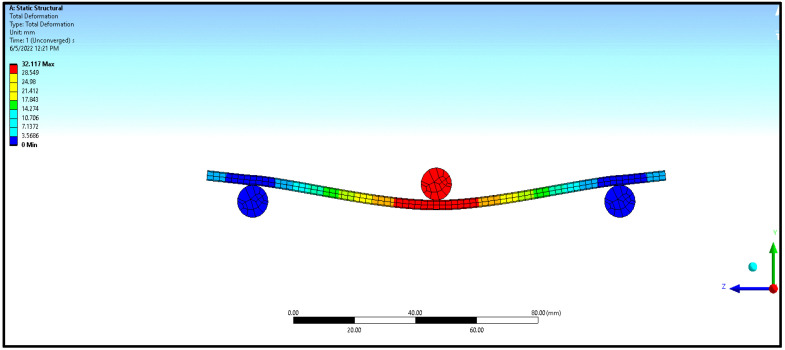
Sample ANSYS output for flexural test and buckling test.

**Figure 10:**
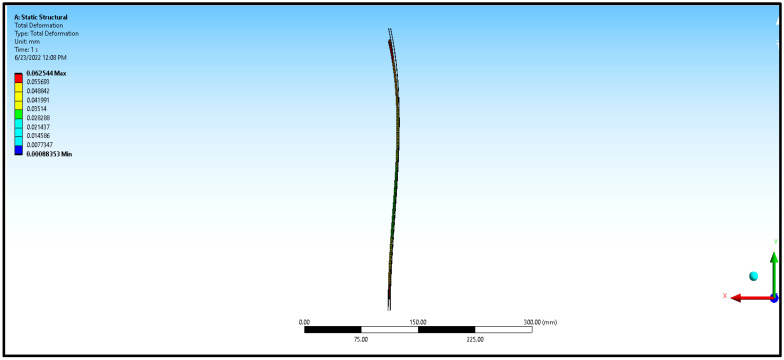
Sample ANSYS output for flexural test and buckling test.

In the numerical analysis, the sandwich composite laminate consisting of high-density polyethylene core was assumed to be a homogeneous material with negligible defects. The maximum bending load was obtained at a lower L/t and a higher b/t, corresponding to 30 and 5, respectively. Improved longitudinal shear stress was the reason for this effect. At a higher L/t, the sandwich composite laminates became very weak against bending due to the accumulation of stress at the contact surfaces between the metal face layer and the core. The stress accumulated on the contact surfaces varied across the span of the laminate. This was confirmed from the observations of the modeled output of the maximum bending load in the range of 0.65 to 0.75 kN at L/t = 43.33 compared to that at L/t = 30, where it ranges between 1 to 1.6 kN. The corresponding maximum deflection complemented the output measure, i.e., the bending load. The deflection measured doubled (~98%) when the aspect ratio, L/t, increased from 30 to 43.33. Thus, the weakening effect of the sandwich laminates at a higher L/t resulted in increased deflection and poor flexural stiffness as well. The results obtained using both numerical and experimental approaches were complementary with respect to each other, with the least significance of error. The errors obtained in the performance measures between the experimental and numerical approaches were less than 10% in most of the trials except for a few. The flexural stiffness of about 0.164 kN/mm for L/t = 30 and b/t = 5 reduced drastically to 0.043 kN/mm for the same b/t and L/t = 43.33. A similar failure phenomenon was observed in the results of inter-laminar shear stress of the sandwich laminate. An almost 50% reduction was seen in the inter-laminar shear stress of the laminate with an increase in the aspect ratio, L/t, from 30 to 43.33. This once again confirmed that the process was consistent and accurate in terms of design and modelling remaining closer to the experimental conditions. The accuracy and precision of the results obtained reflect the fact that the same replications were carried out in the numerical study and experimentation. From the observations of the buckling test shown in Table 2 and Figure 8, it can be gleaned that both the approaches, namely, experimental and numerical, resulted in consistent and complementary data. The maximum critical buckling load was obtained at an aspect ratio, b_3_/t, corresponding to the width of 10 mm of the sandwich specimen. Since the buckling load was compressive in nature, the critical buckling load capacity was greatly influenced by the cross-section of the laminate. Compared to the cross-section of the laminate, the influence of end conditions during the buckling test was less significant. The decrease in the width of the sandwich laminate significantly reduced the buckling load capacity. The greater the width of the laminate, the higher the shear force distribution across the thickness of the laminate, which, in turn, increases the resistance toward lateral deflection and improves buckling performance [24,25]. In the fixed–fixed end condition, the resistance towards buckling was higher compared to that of other end conditions for the same dimensions of the laminate. The constrained moment and reactive forces developed at the ends of the laminate resulted in weaker slenderness and stronger buckling. The equivalent system of forces for the buckling load tended to fall in the failure region for a minimum cross-sectional area. The results obtained through the experimental and numerical approaches significantly demonstrated the fact that the approximated numerical models for bending and buckling behaviors were precise enough, with consistent results and the least possibility of errors. The sample output of numerical analysis for bending and buckling was shown in Figure 9 and Figure 10.

## 4. Conclusions

Aluminum sandwich composite laminates (ASCs) were tested for their flexural and buckling behavior, and the following conclusions were drawn from the study.

(i) The influence of aspect ratios, i.e., the support-span-to-thickness and width-to-thickness ratios, on the flexural and buckling behavior of the sandwich composite laminate was significant.
(ii) The observations obtained from the flexural test revealed that the aspect ratios, L/t and b/t, influenced the laminate’s flexural stability significantly. Though the adhesive layer connecting the metal face layer and the core contributed less to the bending behavior of the laminate, it significantly affected the overall ductility of the laminate.
(iii) Critical bending load and flexural stiffness were maximum for the support span with a width of 90 mm and 15 mm, corresponding to 3.6 kN and 4.75 kN/mm, respectively. The resistance offered against bending was maximum at a greater width. Similarly, with a higher support span, the spring-back effect was reduced, resulting in large-scale bending.
(iv) A higher magnitude of inter-laminar shear stress was noticed for the widths 10 mm and 15 mm, whereas it was minimum for the width of 12 mm. Hence, it was found that the optimum width of this sandwich laminate with a length of 150 mm was 12 mm in order to resist the delamination shear of the laminate.
(v) Maximum critical buckling load was obtained for the aspect ratio b_3_/t, corresponding to the width of 10 mm of the sandwich specimen, where the contribution towards the buckling resistance was high.
(vi) The results obtained from the bending and buckling behavior of the aluminum–polyethylene sandwich laminate reveal that these kinds of panels perform better in design and stability for lower-altitude structures than for higher-elevated structures. A lower aspect ratio (span to thickness) benefited these sandwich laminates to a greater degree in terms of both bending and buckling.

## Figures and Tables

**Table 1:** Numerical vs. experimental results.

S. No.	L/t	b/t	Maximum Bending Load (kN)			Maximum Deflection (mm)			Flexural Stiffness (kN/mm)			Inter-Laminar Shear Stress (kN/mm^2^)		
			Expt.	Num.	Error (%)	Expt.	Num.	Error (%)	Expt.	Num.	Error (%)	Expt.	Num.	Error (%)
1	30	5	1.34	1.63	17.3	10.19	9.91	2.7	0.13	0.16	−23.1	0.022	0.027	−22.7
2	30	4	1.02	1.26	18.4	9.71	9.93	−2.3	0.10	0.12	−20.0	0.021	0.026	−23.8
3	30	3.3	1.00	1.01	1.1	11.35	8.42	25.8	0.08	0.12	−50.0	0.025	0.026	−4.0
4	36.67	5	0.75	0.78	4.8	10.36	13.59	−31.2	0.07	0.05	28.6	0.012	0.013	−8.3
5	36.67	4	0.62	0.80	22.7	10.73	13.12	−22.3	0.05	0.06	−20.0	0.012	0.017	−41.7
6	36.67	3.3	0.51	0.61	15.4	10.72	12.97	−21.0	0.04	0.04	0.0	0.012	0.015	−25.0
7	43.33	5	0.75	0.84	10.8	17.24	19.67	−14.1	0.04	0.04	0.0	0.012	0.014	−16.7
8	43.33	4	0.77	0.78	1.1	22.11	19.87	10.1	0.03	0.04	−33.3	0.016	0.016	0.0
9	43.33	3.3	0.65	0.75	13.3	22.27	19.31	13.3	0.02	0.03	−50.0	0.016	0.019	−18.8

**Table 2:** Numerical vs. experimental buckling load analysis results.

S. No.	End Condition	b/t	Bucking Load (N)		
			Num.	Expt.	Error (%)
1	1	5	155	158	1.9
2	1	4	116.2	122	4.8
3	1	3.3	101.9	99.78	−2.1
4	2	5	342	346.4	1.3
5	2	4	221	219.73	−0.6
6	2	3.3	204.6	206.9	1.1
7	3	5	655.8	654.8	−0.2
8	3	4	524.8	526.77	0.4
9	3	3.3	422	424.7	0.6

## Data Availability

The data that support the findings of this study are available within the article.
